# Supplementary material for: Genomic and machine learning approaches to predict antimicrobial resistance in Stenotrophomonas maltophilia
Source: Microbiol Spectr. 2025 Jun 18;13(8):e02632-24. doi: 10.1128/spectrum.02632-24 (PMC12323353; doi:10.1128/spectrum.02632-24)
Supplement: Figure S1 — Impact of Sample Size on LEV and SXT Resistance Model Performance. [file spectrum.02632-24-s0001.docx]

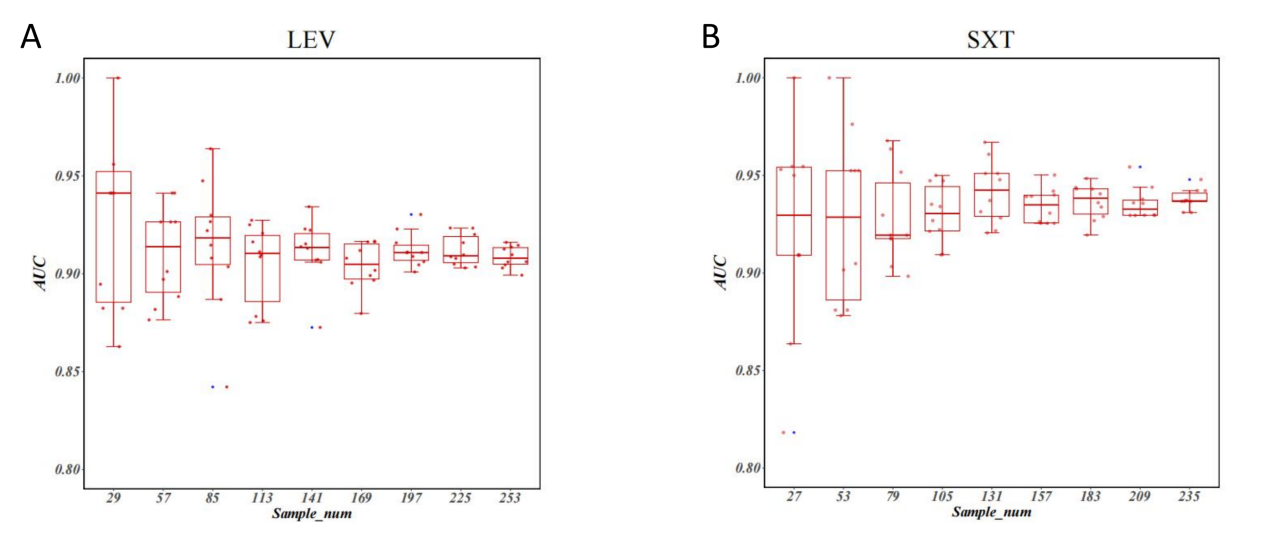


**Figure S1: Impact of Sample Size on LEV and SXT Resistance Model Performance.** Evaluation of the effect of different sample sizes on the model's AUC. The x-axis represents 10-90% of the sample size, and the y-axis represents the AUC of the model. (A) Curve showing the impact of sample size on the LEV resistance model AUC; (B) Curve showing the impact on the SXT resistance model AUC.
