# Supplementary material for: Genomic and machine learning approaches to predict antimicrobial resistance in Stenotrophomonas maltophilia
Source: Microbiol Spectr. 2025 Jun 18;13(8):e02632-24. doi: 10.1128/spectrum.02632-24 (PMC12323353; doi:10.1128/spectrum.02632-24)
Supplement: Supplemental material — Graphical abstract. [file spectrum.02632-24-s0002.pdf]

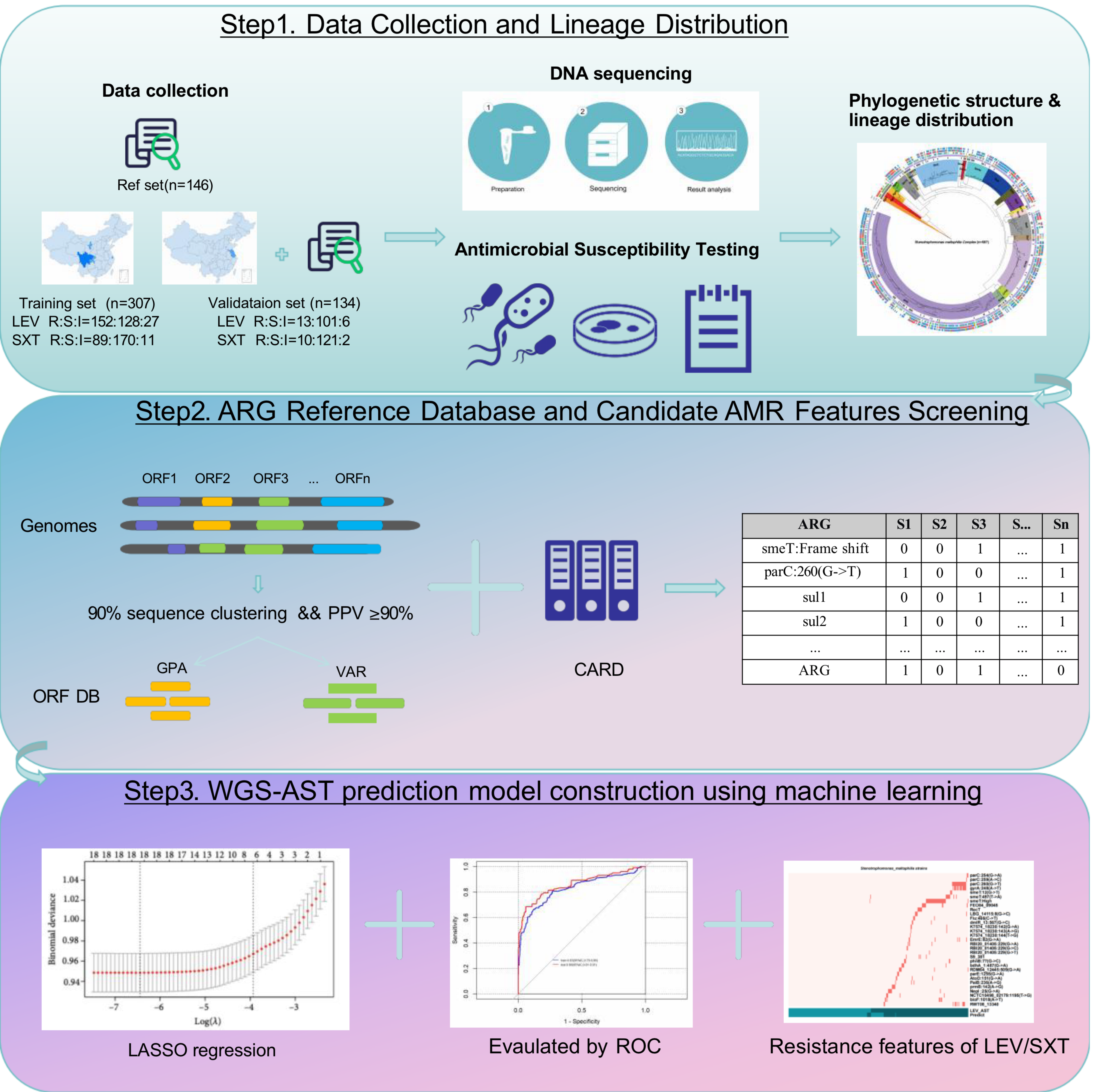

Step1. Data Collection and Lineage Distribution
Data collection
Ref set(n=146)
Training set (n=307)
LEV R:S:I=152:128:27
SXT R:S:I=89:170:11
Validataion set (n=134)
LEV R:S:I=13:101:6
SXT R:S:I=10:121:2
DNA sequencing
Preparation
Sequencing
Result analysis
Antimicrobial Susceptibility Testing
Phylogenetic structure & lineage distribution
Step2. ARG Reference Database and Candidate AMR Features Screening
ORF1 ORF2 ORF3 ... ORFn
Genomes
90% sequence clustering && PPV ≥90%
GPA
VAR
ORF DB
CARD
ARG | S1 | S2 | S3 | S... | Sn
smeT:Frame shift | 0 | 0 | 1 | ... | 1
parC:260(G->T) | 1 | 0 | 0 | ... | 1
sul1 | 0 | 0 | 1 | ... | 1
sul2 | 1 | 0 | 0 | ... | 1
... | ... | ... | ... | ... | ...
ARG | 1 | 0 | 1 | ... | 0
Step3. WGS-AST prediction model construction using machine learning
Binomial deviance
Log(λ)
LASSO regression
Sensitivity
1 - Specificity
Evaulated by ROC
Resistance features of LEV/SXT
